# Distinctive Imaging Characteristics of Retinal and Cerebral Vessels between Central and Branch Retinal Vein Occlusion by MRI and AI-Based Image Analyzer

**DOI:** 10.3390/diagnostics14030267

**Published:** 2024-01-26

**Authors:** Qiyun Wang, Ting Li, Xinyuan Zhang, Yiyun Zeng, Yang Yang, Yun Zhou, Xinming Gu, Xiaobin Xie, Saiguang Ling

**Affiliations:** 1 Beijing Tongren Eye Center, Tongren Hospital, Capital Medical University, Beijing 100730, China; tryywqy2024@163.com (Q.W.); trykzyy@163.com (Y.Z.); tiffafighting@163.com (Y.Y.); 18812566649@163.com (X.G.); 2 Beijing Retinal and Choroidal Vascular Disorders Study Group, Tongren Hospital, Capital Medical University, Beijing 100730, China; 3 Department of Radiology, Capital Medical University, Beijing Tongren Hospital, Beijing 100176, China; bgpliting2004@126.com; 4 China National Clinical Research Center for Neurological Diseases, Tiantan Hospital, Capital Medical University, Beijing 100730, China; anmui@sina.com; 5 Eye Hospital of China Academy of Chinese Medical Sciences, Beijing 100040, China; xiexiaobin0622@163.com; 6 EVision Technology, Beijing 100085, China; lingsaiguang@yiweiimage.com

**Keywords:** retinal vein occlusion, geometric characteristics, geometric characteristics of the retinal vessels, MRI, primary open-angle glaucoma, optic nerve subarachnoid space

## Abstract

Retinal vessels have been good predictive and prognostic imaging biomarkers for systemic or eye diseases. Numerous studies have shown that the two retinal vein occlusion entities may correlate with cardiovascular and cerebrovascular events or primary open-angle glaucoma. This study aims to investigate if there is a disparity in the correlations between branch RVO (BRVO) and central RVO (CRVO) with systemic disorders or POAG, thus explaining the pathogenic difference between BRVO and CRVO. This retrospective case-control study enrolled 59 RVO subjects (118 eyes), including 25 CRVO and 34 BRVO subjects, who received routine eye and brain MRI examinations. The geometric characteristics of the caliber of the retinal and cerebral blood vessels and the optic nerve subarachnoid space width (ONSASW) were measured. Multivariable logistic regression analysis showed that ONSASW at 3 mm behind the globe (*p* = 0.044) and the relative retinal venular calibers (*p* = 0.031) were independent risk factors for the CRVO-affected eyes group in comparison with the BRVO-affected eyes group after adjusting for age, duration of hypertension, BMI, and IOP. In the CRVO-affected eyes, narrower relative retinal arteriolar calibers (*p* = 0.041) and wider relative venular calibers (*p* = 0.011) were independent risk factors compared with the CRVO-contralateral normal eyes when adjusting for IOP. We concluded that BRVO may be more associated with cerebrovascular diseases, and CRVO may be correlated with primary angle glaucoma. The geometric characteristics difference between the retinal and cerebrovascular may explain the pathological difference between CRVO and BRVO.

## 1. Introduction

Retinal vein occlusion (RVO) is the most common visual impairment retinal vascular disease after diabetic retinopathy [1]. RVO was broadly classified as central retinal vein occlusion (CRVO), hemispheric retinal vein occlusion (HRVO), which is considered a variant of CRVO (a congenital anomaly of CRVO) [2] or branch retinal vein occlusion (BRVO). RVO can also be ischemic or non-ischemic RVO according to the perfusion status of the retina.

Some traditional risk factors for cardiovascular and cerebrovascular diseases, such as a thrombotic event, cigarette smoking, high activated factor VII, dyslipidemia, high blood viscosity, older age, hypertension, carotid artery, and cerebrovascular diseases, were associated with the occurrence, severity, or secondary macular edema of RVO [3,4]. However, these risk factors are insufficient to fully explain the arterial thromboembolic events, especially the distinct disparity of these two phenotypes of RVO, which remains unclear, and the pathophysiology of RVO is still elusive.

Primary open-angle glaucoma (POAG) has been implicated in the pathogenesis of RVO. However, existing studies did not provide consistent results, and a conclusive link remains elusive [2]. POAG (odd ratio, OR: 5.03, 95% confidential: 3.97–6.37) was found to be a significant risk factor for RVO, and the risk for CRVO (odd ratio, OR: 5.3) was significantly higher than BRVO (OR: 0.65) in an advanced subgroup analysis of a meta-analysis [5], indicating POAG may have a closer relationship with CRVO than BRVO. It was reported that POAG with normal pressure may have an abnormally low cerebrospinal fluid pressure (CSF-P) in the retrobulbar space; the narrower orbital optic nerve subarachnoid space (ONSASW) in patients with POAG was correlated with normal intraocular pressure; and a lower orbital CSF-P in patients with POAG was associated with normal intraocular pressure [6].

Previous studies also found that BRVO and CRVO correlate with cardiovascular and cerebrovascular events, including stroke, myocardial infarction, and arterial stiffness. Furthermore, the incidence risk is insistent [7,8,9]. It was also reported that CRVO is associated with a higher prevalence of diabetes mellitus and chronic kidney disease due to elevated homocysteine levels [10].

Although these two RVO entities share some common risk factors, especially systemic factors, it is also critical to realize that BRVO and CRVO are otherwise distinct entities, necessitating various management approaches and producing various issues.

Retinal vessels have been good predictive and prognostic imaging biomarkers for systemic or eye diseases. Numerous studies have shown that the two retinal vein occlusion entities may correlate with cardiovascular and cerebrovascular events or primary open-angle glaucoma. This study aims to investigate if there are disparities in the correlations between BRVO and CRVO with systemic disorders or POAG using the retinal vessel geometric characteristics (RVGCs) and cerebral blood vessel geometric characteristics (CBVGCs), as well as the ONSASW. We further discuss if the difference in imaging characteristics could explain the pathogenic disparity between BRVO and CRVO.

## 2. Materials and Methods

### 2.1. Participants

In this retrospective case-control study, 59 patients (118 eyes) with one eye being naïve RVO and the contralateral eye being normal were randomized and enrolled from February 2018 to August 2019 in the outpatient clinic of Beijing Tongren Hospital. The contralateral normal eyes of the BRVO and CRVO were registered as the control eyes. This study was approved by the Ethics Committee of Beijing Tongren Hospital (TREC2023-KY018 and TRECKY2016-054-XZ-1); all subjects were assigned informed consent before enrollment, and the study was conducted according to the tenets of the Declaration of the Helsinki-Ethical principle for medical research involving human subjects.

The inclusion criteria were: (1) naïve RVO not treated with anti-vascular endothelial growth factor (VEGF) at least within one year; and (2) the contralateral eyes were normal. The diagnostic criteria for RVO were according to “Retinal Vein Occlusions Preferred Practice Pattern^®^ (PPP) 2019” by the American Academy of Ophthalmology [11]. The diagnosis of BRVO or CRVO was based on the patient’s medical history, a detailed eye examination, and a diagnostic tests described as follows: The exclusion criteria were: (1) subjects with bilateral retinal, choroidal diseases; (2) patients with other retinal vascular diseases or eye diseases, such as hypertensive retinopathy, diabetic retinopathy, macular degeneration, Eale’s disease, etc.; (3) patients with a recent surgical history on one of the eyes; (4) patients with severe systemic disease, such as stroke and cerebrovascular diseases, within six months and were unable to tolerate eye examinations.

Fundus ophthalmoscopy and optical coherence tomography (OCT) configuration evaluated the contralateral normal fundus control. An experienced retinal specialist determined the patients’ enrollment.

All the enrolled eyes were further grouped into the CRVO-affected eyes group, the CRVO-contralateral normal eyes group, the BRVO-affected eyes group, and the BRVO-contralateral normal eyes group.

### 2.2. Eye Examination

Patients with RVO were evaluated in all relevant aspects. All enrolled subjects underwent complete eye examinations including refractometry, best corrected visual acuity, non-contact intraocular pressure (TX20 Automatic Non-contact Tonometer, Canon Co., Ltd., Tokyo, Japan), slit-lamp microscopic examination (SL-IE Slit Lamp Microscope, Topcon Co., Ltd., Tokyo, Japan), pupillary assessment, Gonioscopy assessment, binocular funduscopic evaluation of the posterior pole, indirect ophthalmoscope (to examine the peripheral retina and vitreous, signs of ischemia including neovascularization of the disc or elsewhere, etc.), fundus fluorescein angiography including Red-free fundus photography (Heidelberg Retina Angiograph II, Heidelberg Engineering, Heidelberg, Germany) and fundus photography (CR-1 non-mydriatic Fundus Camera, Canon Co., Ltd.). OCT (DRI OCT1 Atlantis scanner, Topcon Co., Ltd., Tokyo, Japan; PLEX^®^ Elite 9000, Carl Zeiss Meditec Inc., Dublin, CA, USA) and OCT angiography.

Systemic examinations were performed for all enrolled subjects, including blood pressure and routing fasting biochemical examinations. Baseline information about age, medical history (duration of hypertension), body weight, height, and body mass index (BMI) was collected.

#### 2.2.1. AI-Based Image Analyzer for Quantitative Measurement of the Relative Retinal Vessel Caliber

**Figure 1:**
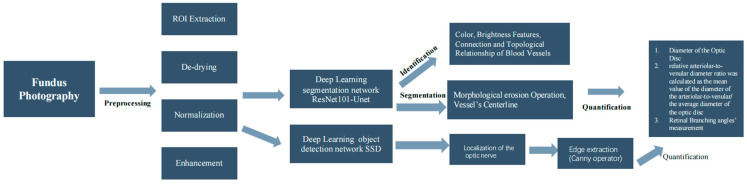
The flow chart illustrates the working procedure of the AI-based image analyzer (EVision).

**Figure 2:**
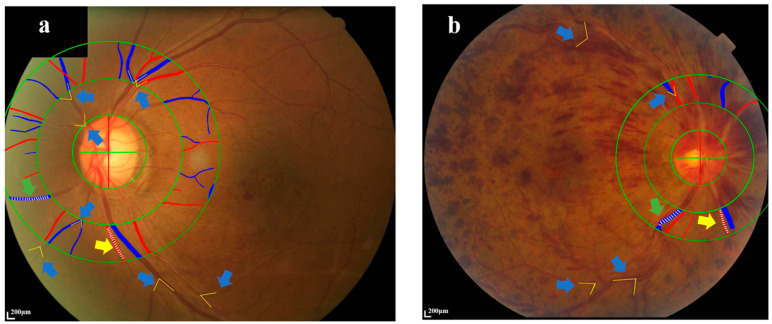
AI-based image analyzer (EvisionAI, Beijing, China) quantifies the retinal blood vessel geometric parameters in the RVO-affected eyes. (**a**) showed the measurement in the BRVO-affected eye. (**b**) showed the measurement in the CRVO-affected eye. The yellow grottos pointed by the blue arrows in (**a**,**b**) represent the first angle at the vascular bifurcation. The relative arteriolar orvenular diameter was calculated as the mean value of the diameter of the arteriolar or venular/the average diameter of the optic disc. Green arrow: retinal vein. Yellow arrow: retinal artery. Green circles: the radius of these circles is 0.5 DD, 1 DD and 1.5 DD at the center of the optic disk respectively. BRVO: Branch retinal vein occlusion. CRVO: Central retinal vein occlusion. AI: Artificial intelligence. DD: the diameter of the optic disc.

After pharmacological pupil dilation, Fundus photography with 45° centered on the optic disc and macular was taken using a Canon retinal camera. Fundus image AI-based software EVision [12,13] was used to intelligently identify fundus blood vessels from fundus images and automatically identify and mark the margin of the optic disc with manual corrections to calculate the diameter of blood vessels. The software integrates artificial intelligence image processing technologies such as computer vision and deep learning (Figure 1). For the fundus image to be processed, it performs four preprocessing operations: ROI extraction, de-drying, normalization, and enhancement to remove the non-fundus structure area, reduce image noise, increase the sharpness of image feature edges, and reduce the difference between images [14,15]. The deep learning segmentation network model ResNet101-Unet was used for retinal vascular segmentation. After obtaining the segmentation results of the blood vessels, based on the features of color, brightness, and the connection and topological relationship of the blood vessels in the fundus, arterial and venous blood can be distinguished and identified. For optic disc segmentation, the deep learning object detection network SSD was used for localization of the optic disc, then the fundus image was transformed in polar coordinates, and the edge extraction algorithm based on the Canny operator was used to obtain the edge of the optic disc in polar coordinate and realize the fine segmentation of the optic disc [12]. Based on the retinal vessel segmentation results of the fundus, the morphological erosion operation is performed on it to obtain the vessel’s centerline. An orthogonal straight-line tangent to a point on the centerline intersects two points on the edge of the vessel, and the distance between the two points is the centerline. The vessel diameter corresponds to this point. Based on the results of optic disc segmentation, the diameter of the smallest circumscribed circle of the optical disc was taken as the diameter of the optic disc (DD), and the average diameter of arteries and veins within 1.0–1.5 DD from the center of the optic disc was calculated. A pixel density histogram marked the boundaries. The retinal arterioles and venules were outlined in different colors and measured (the green and yellow arrows, Figure 2). All traced arterioles and venules were divided into at least ten sections, and the relative arteriolar-to-venular diameter ratio was calculated as the mean value of the diameter of the arteriolar-to-venular/the average diameter of the optic disc [16,17].

#### 2.2.2. Measurement of Retinal Branching Angles

The retinal vascular branching angles were defined as the first angle subtended between two branch vessels at each vascular bifurcation and were evaluated using ImageJ software1.48 (National Institutes of Health, USA) [18] (the blue arrows, Figure 2). The retinal vascular branching angles in the retinal superior temporal artery, inferior temporal artery, superior temporal vein, inferior temporal vein, superior nasal artery, inferior nasal artery, superior nasal vein, and inferior nasal vein were calculated and statistically analyzed.

### 2.3. Cerebrovascular Evaluation

#### 2.3.1. Measurement of Cerebral Blood Vessel Geometric Parameters

A 3.0 TESLA scanner (Signa HDx; General Electric Medical Systems, Milwaukee, WI, USA) using an 8-channel head coil for an MRI of the brain was applied in this study. The three-dimensional time-of-flight magnetic resonance angiography (TOF-MRA) was performed on patients in a supine position; the parameters were set to TR = 28.0 ms, TE = 2.7 ms, flip angle = 20°, slice thickness = 1.2 mm, FOV = 220 mm × 220 mm, matrix = 512 × 512, voxel size = 0.4 × 0.4 × 0.6. All subjects were asked to stare at the target directly attached to the MRI scanner gantry to avoid artifacts due to eye movement. If motion artifacts were detected, the sequence needed to be repeated. Morphological evaluations of the brain vessels were performed independently by two experienced TLs and YZs using the Radiant DICOM Viewer 4.0.2 software at different times. The diameter of the OA, ICA-C6 in the coronal plane, and the average of three measurements were investigated. A professional neurologist diagnosed the MRI images of the brains of all subjects.

#### 2.3.2. Measurement of the Optic Nerve Sheath and Optic Nerve Diameter, and ONSASW

**Figure 3:**
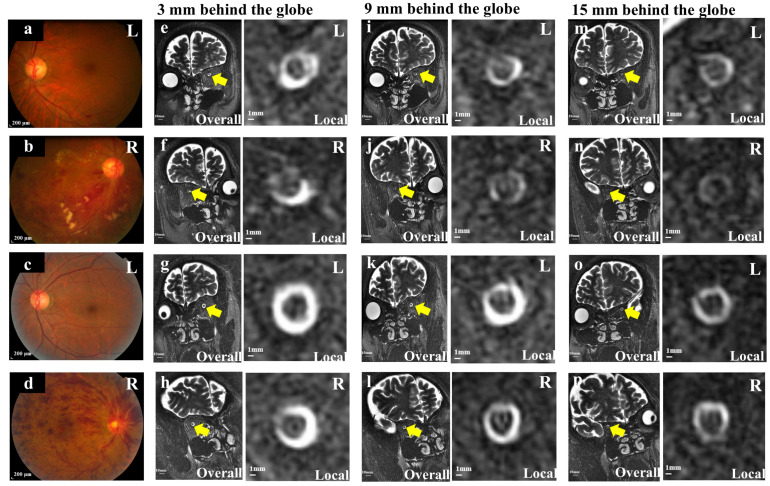
MRI images showed the ONSASW 3 mm, 9 mm, and 15 mm behind the globe in RVO eyes (color photos). (**a**,**b**): The color fundus photography of BRVO-affected or contralateral normal eyes. (**c**,**d**): The color fundus photography of CRVO-affected or contralateral normal eyes. (**e**–**p**) showed an oblique coronal T2WI-FRFSE image with fat suppression to demonstrate the optic nerve sheath complex taken at 3 mm (**e**–**h**), 9 mm (**i**–**l**), and 15 mm (**m**–**p**) behind the globe. (**a**,**b**,**e**,**f**,**i**,**j**,**m**,**n**): A 49-year-old man with BRVO and contralateral normal eyes. (**c**,**d**,**g**,**h**,**k**,**l**,**o**,**p**): A 51-year-old man with CRVO and contralateral normal eyes. Yellow arrow: ONSASW. BRVO: Branch retinal vein occlusion. CRVO: Central retinal vein occlusion. ONSASW: Optic nerve subarachnoid space width.

As described previously [6], to measure the optic nerve/sheath complex, we used the T2-weighted fast recovery fast spin echo (FRFSE) sequence with or without fat suppression to acquire images for both eyes (TR = 6000 ms, TE = 245 ms, ETL = 60, FOV = 1600 mm × 1600 mm, matrix = 320 × 320, NEX = 2, slice thickness = 3 mm, spacing = 3 mm, bandwidth = 20.83 Hz, flip angle = 90°). Three oblique coronal sections perpendicular to the optic nerve sheath complex were selected and evaluated for 3, 9, and 15 mm at the retrobulbar (Figure 3). All morphologic image evaluations were independently performed by two experienced observers on the same post-processing software 3.0 (Signa HDx; General Electric Medical Systems, Milwaukee) in a masked manner. The images were viewed at 2000 Hounsfield units (HU) window width and 1000 HU window level. The horizontal and vertical sections of the optic nerve (OND) and optic nerve sheath (ONSD) were also measured, and the mean values were recorded, respectively. The ONSASW was calculated by subtracting half the diameter of the optic nerve from half of the diameter of the optic nerve sheath based on formulae as described by Wang et al. [6].

### 2.4. Sample Size Determination

We determined the sample size using the Power Analysis and Sample Size 15 software. According to our pilot study, at a 95% confidence level with a margin of error of +/−5% to detect the difference (0.9) between the groups.

### 2.5. Statistical Analysis

SPSS software (SPSS, Inc. 23.0, Chicago, IL, USA) was applied for the statistical analysis. Data normality was evaluated by the Kolmogorov-Smirnov test and the Shapiro-Wilk test. Baseline demographics of the patients and imaging parameters were described as means ± standard deviation (means ± SD) or medians (interquartile ranges, IQR). We tested the homogeneity of the variance using Levene’s test. One-way analysis of variance (ANOVA), the chi-square test, the independent sample *t*-test, and the Mann-Whitney U test were used to compare the differences among the BRVO or CRVO groups. A paired sample *t*-test was used to compare the difference between RVO-affected and contralateral normal eyes. Multiple-logistic regression models were used to compare the group’s differences in the imaging markers. The odds ratio (OR) was adjusted for age, BMI, duration of hypertension (HBP), and intraocular pressure (IOP). As we described, when the contralateral normal eyes are the reference, OR > 1 means the variable is an independent risk factor for the disease (BRVO or CRVO), and OR < 1 means the variable is a protective factor for the disease (BRVO or CRVO). When group comparison between BRVO- and CRVO-affected eyes occurs, OR > 1 means the variable is an independent risk factor for the study eyes, and OR < 1 means the variable is a protecting factor for the study eyes. *p* < 0.05 indicated statistical significance [19].

## 3. Results

### 3.1. Baseline Demographic and Clinical Characteristics

Fifty-nine subjects (118 eyes) had an average age of 52 ± 2 years. BRVO included 34 subjects (68 eyes) with an average age of 53 ± 10 years. CRVO included 25 subjects (50 eyes) with an average age of 50 ± 15 years. Patients with CRVO were eligible to enroll in this study. The contralateral normal eyes were also registered as the control (Table 1).

**Figure 4:**
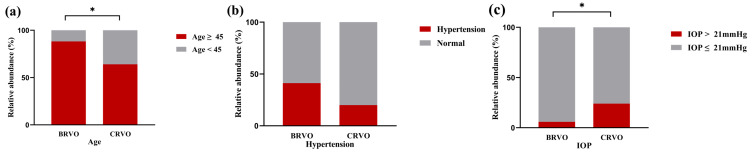
Comparisons of the baseline characteristics between BRVO and CRVO groups. A Chi-squared test was used to compare proportions of age (**a**), hypertension (**b**), and IOP (**c**). * Statistically significant: *p* < 0.05. BRVO: Branch retinal vein occlusion. CRVO: Central retinal vein occlusion. IOP: Intraocular pressure.

No statistically significant differences in age, gender, or BMI were found between the subjects with BRVO and CRVO (*p* > 0.05) (Table 1). In subgroup analysis, the proportion of patients above 45 years with BRVO was significantly higher than that of the CRVO patients (88.2% vs. 64.00%, *p* = 0.026). IOP in the CRVO-affected eyes was significantly higher than in the BRVO-affected eyes (*p* = 0.024) (Figure 4). The duration of HBP in patients in the BRVO group is significantly higher than that in the CRVO subjects, with statistical significance (*p* = 0.022).

### 3.2. Cerebral Blood Vessel Geometric Characteristics in Comparison with the BRVO and CRVO Groups

The diameter of the OA in the BRVO-contralateral normal eyes was significantly narrower than that in the CRVO-contralateral normal eyes (*p* = 0.014). There was no statistical significance in the diameter of ICA-6 between the four groups (*p* > 0.05) (Table 2).

All subjects’ brain MRI images were screened and evaluated by an experienced neurologist (20 years of working experience). The qualitative MRI imaging analysis showed that the proportion of the reduced number of the middle cerebral artery and its branches in the affected eyes and narrower vascular lumens and stiffened shapes of the lumens was significantly higher in the BRVO group (10/34, 29%) than that in the CRVO group (29% vs. 56%) (*p* = 0.040).

### 3.3. Comparison of ONSASW between the Groups

**Figure 5:**
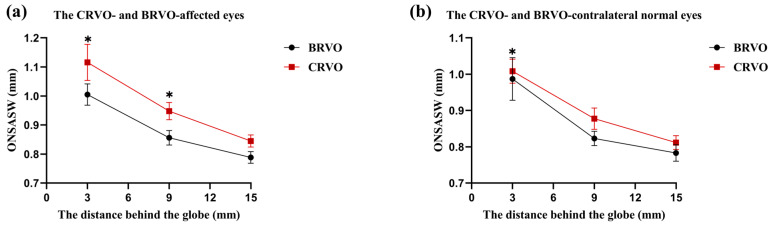
Comparisons of ONSASW at the distances of 3 mm, 9 mm, and 15 mm behind the globe between the CRVO- and BRVO-affected eyes and the CRVO- and BRVO-contralateral normal eyes. Group comparisons were analyzed by one-way analysis of variance (ANOVA). * Statistically significant: *p* < 0.05. BRVO: Branch retinal vein occlusion. CRVO: Central retinal vein occlusion. ONSASW: Optic nerve subarachnoid space.

In a previous study, ONSASW measurement was an invasive method for reflecting the cerebrospinal fluid pressure (CSF-P) in the retrobulbar space; POAG is associated with a narrower space of the ONSASW in the CRVO-affected eyes; the ONSASW at 3 mm (*p* = 0.003) and 9 mm (*p* = 0.015) behind the globe was significantly wider than that of the contralateral normal eyes. The ONSASW at 3 mm (*p* = 0.001) and 9 mm (*p* = 0.022) behind the globe of the CRVO-affected eyes group was significantly wider than that of the BRVO-affected eyes group. The ONSASW at 3 mm behind the globe of the CRVO-contralateral normal eyes group was significantly wider than that of the BRVO-contralateral normal eyes (*p* = 0.029) (Figure 5; Table 2).

**Figure 6:**
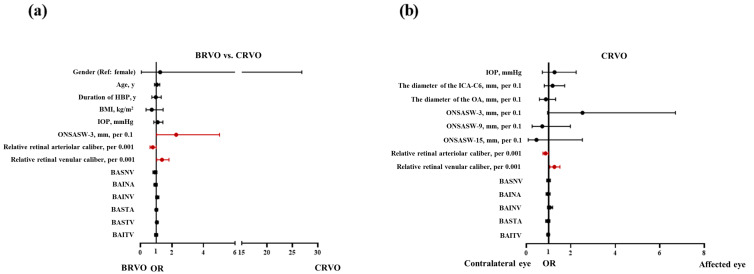
The multivariate multiple-logistic regression analysis shows that (**a**) ONSASW at 3 mm and wider relative retinal venular caliber were risk factors for CRVO compared to BRVO. In contrast, the relative retinal arteriolar caliber was a risk factor for BRVO compared with CRVO. (**b**) Narrower relative retinal arteriolar and wider relative venular calibers in affected eyes were risk factors compared with contralateral normal eyes in CRVO. Statistically significant: *p* < 0.05. BRVO: Branch retinal vein occlusion. CRVO: Central retinal vein occlusion. HBP: high blood pressure. BMI: Body mass index. IOP: Intraocular pressure. ONSASW: Optic nerve subarachnoid space. BASNV Branching angles in a retinal superior nasal vein. BAINA: Branching angles in a retinal inferior nasal artery. BAINV: Branching angles in a retinal inferior nasal vein. BASTA: Branching angles in a retinal superior temporal artery. BASTV: Branching angles in a retinal superior temporal vein. BAITV: Branching angles in a retinal inferior temporal.

Multiple-logistic regression analysis showed that ONSASW at 3 mm behind the globe (*p* = 0.044) and the relative retinal venular calibers (*p* = 0.031) were independent risk factors for CRVO-affected eyes in comparison with the BRVO-affected eyes when adjusting for age, duration of hypertension, BMI, and IOP (Figure 6a).

### 3.4. Retinal Vessel Geometric Characteristics in the BRVO and CRVO Groups

The relative retinal venular caliber and AVR between the affected and the contralateral normal eyes in the CRVO group had statistical differences (*p_all_* < 0.01). However, no statistical differences existed between the affected and the contralateral normal eyes in the BRVO group, relative retinal venular caliber, or AVR (*p* > 0.05). The relative retinal venular caliber and AVR in the BRVO-affected eyes were significantly different from those in the CRVO-affected eyes (*p_all_* < 0.01) (Table 2).

Multivariate logistic regression analysis showed that in the CRVO-affected eyes, narrower relative retinal arteriolar (*p* = 0.041) and wider relative venular calibers (*p* = 0.011) were risk factors compared with the contralateral normal eyes when adjusting for IOP (Figure 6b).

There was no difference in the retinal vascular branching angles of the superior nasal artery, superior temporal artery, and inferior temporal artery between the BRVO-affected eyes group and the contralateral normal eyes group (*p* > 0.05). In comparison with the CRVO-affected eyes group, the retinal vascular branching angles of the superior nasal artery (*p* < 0.01), superior nasal vein (*p* = 0.032), inferior nasal artery (*p* = 0.020), superior temporal artery (*p* = 0.011), and inferior temporal artery (*p* = 0.002) were significantly larger in the BRVO-affected eyes group. There is no difference between the two groups compared to the contralateral normal eyes of the BRVO and CRVO eyes (*p* > 0.05) (Table 2).

**Figure 7:**
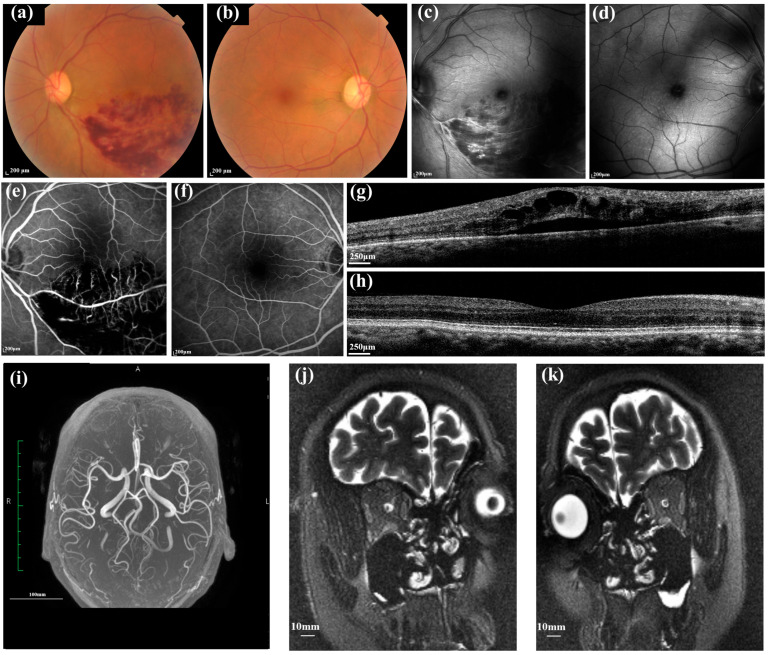
Advanced multimodal images of a 62-year-old female with BRVO in the left and normal contralateral right eyes. The best corrected visual acuity was 20/70 in the left eye and 20/30 in the right eye. Intraocular pressure was 18 mmHg in both eyes. Color fundus photos showed diffuse hemorrhages in the inferior temporal quadrant in the left eye (**a**), and the right eye looks normal (**b**). Infrared images of the left (**c**) and right eye (**d**). A late-phase frame of the fluorescein angiogram demonstrates dilated, tortuous veins with the area of fluorescein blockage in the left eye (**e**); the right eye looks normal (**f**). OCT image of the left eye shows the intra- and sub-retinal fluid and hyper-reflective foci in the inner retina (**g**), and the macular structure in the right eye is normal (**h**). The MRI imaging showed a reduced number of the middle cerebral artery and its branches in the affected left eye (**i**). The ONSASW behind the bilateral globe showed no difference using the MRI ((**j**,**k**); oblique coronal T2WI-FRFSE image with fat suppression). The advanced stage of BRVO, complicated by macular edema, showed that the retinal vessel was intensively affected, which correlates with impaired cerebral vessels. This case also showed that the ONSASW did not significantly differ from the contralateral eye, further supporting the hypothesis that BRVO may have a close relationship with cerebrovascular events, not glaucoma. BRVO: Branch retinal vein occlusion. ONSASW: Optic nerve subarachnoid space width. OCT: Optical coherence tomography.

Figure 7 shows imaging characteristics of the retinal and cerebral vessels in patients with BRVO in the left eye and contralateral normal eye using MRI and an AI-based image analyzer.

## 4. Discussion

This study found that the proportion of patients over 45 years old with BRVO was significantly higher than that of the CRVO group. The duration of HBP was significantly longer in BRVO patients than in CRVO subjects. The IOP in the CRVO-affected eyes group is significantly higher than in the BRVO-affected eye group. Multiple-variable regression analysis showed that, compared with the BRVO-affected eyes group, the ONSASW at 3 mm and 9 mm behind the globe was significantly wider than that in the CRVO-affected eyes group. The retinal vascular branching angles of the superior nasal artery, superior nasal vein, inferior nasal artery, superior temporal artery, and inferior temporal artery were substantially narrower in the CRVO-affected eyes group. All the above results indicated different pathophysiological mechanisms underlying the two phenotypes of RVO.

As early as 1975, the anatomical and physiological homologies between retinal and cerebral microvasculature had been described; retinal microvascular abnormalities are markers of cerebral microangiopathy [18]. As useful imaging biomarkers, retinal vascular geometry characteristics can reflect microvascular pathological changes, predicting the occurrence of retinal diseases and systemic diseases, including diabetes, hypertension, subclinical cerebral infarcts, and stroke [17,20,21]. A prospective cohort study, the Multi-Ethnic Study of Atherosclerosis (MESA), has reported that stroke can be predicted by retinal arteriolar caliber [22]. Retinal microvascular changes, significantly narrower retinal arterioles, were associated with cerebral microvascular disease or atherosclerosis; lumen diameters are sensitive responses to endothelial dysfunction from inflammation, changes in blood flow under tissue hypoxia, and other physiologic changes [21,23]. A meta-analysis found that patients with RVO increased their risk by 50% for cerebrovascular diseases and 16.8% for stroke in a population-based study [8,24]. However, these studies did not discuss the correlation between subtypes of RVO and cerebrovascular or cardiovascular diseases. Since most of the risk factors for BRVO contribute to the pathogenesis of cerebrovascular diseases [25], it is interesting to further explore the imaging biomarkers of BRVO for predicting cerebrovascular or cardiovascular diseases.

This study found that the OA, the relative vein and arteriolar caliber, and the branch vein or arteriolar angles were differentially associated with BRVO and CRVO. Narrower OA and the relative retinal arteriolar caliber in patients with BRVO increased the risk of cerebrovascular disease, consistent with previous studies [26,27]. In a case series study of comparison of BRVO and CRVO using color Doppler imaging, the parameters of ocular hemodynamics parameters of OA, short posterior ciliary artery (PCA), central retinal artery (CRA), and maximum blood flow velocity (Vmax) in the central retinal vein (CRV) were reduced in the BRVO-contralateral normal eyes than in the normal control eyes. Still, no such results were found in the CRVO-contralateral normal eyes [28]. OA, PCA, and CRA, end-diastolic velocity (PSV), and end-diastolic velocity (EDV) of the BRVO contralateral normal eyes were lower than the normal eyes (*p* < 0.05). We also found that the relative retinal venular caliber of the CRVO-affected eyes was wider than that of the BRVO-affected eyes, suggesting that CRVO or BRVO are not triggered by the same retinal vascular phenotypes that do not trigger CRVO or BRVO. Furthermore, lower AVR in CRVO with an enlargement of the retinal venous network may be due to decreased oxygen saturation by a local variation of the vasodilator [29]. Hardarson et al. found that oxygen saturation in retinal venules was lower in CRVO, but arteriolar saturation is the same in CRVO-affected and contralateral normal eyes [30], which is consistent with our current findings, indicating that the wider relative retinal venular caliber in the CRVO-affected eyes is due to the lower vein oxygen saturation.

We assumed that contralateral normal eyes reflected the phenotype of both eyes. The OA caliber in the BRVO-contralateral normal eyes was found to be significantly narrower than that in the CRVO-contralateral normal eyes, further supporting our hypothesis that BRVO may be more associated with cerebrovascular diseases, which is consistent with the previous studies [26]. OA is the first branch of the internal carotid artery, merging from the cavernous sinus [31]. Retinal blood flow increased after glucose, which has a direct vasodilator action in the contralateral normal eyes of patients with BRVO, indicating that in response to hyperglycemia in RBF might reflect the microvasculature abnormalities, which is consistent with our current findings.

The central retinal vein travels through the center of the optic nerve and the orbital CSF space before exiting the optic nerve, accompanied by the central retinal artery, to drain into the superior ophthalmic vein or the cavernous sinus. The anatomical characteristic suggests that the retinal venous pressure should be at least as high as the orbital cerebrospinal fluid pressure [32]. Wider retinal venular caliber is associated with higher estimated cerebrospinal fluid pressure [33], which implies that CRVO eyes may have higher cerebrospinal fluid pressure. Furthermore, we found that the width of ONSASW at 3 mm and 9 mm in the CRVO-affected eyes behind the globe is wider than that in the BRVO group, suggesting that wider ONSASW is a risk factor for CRVO. The orbital subarachnoid space around the optic nerve is continuous with the cerebrospinal fluid (CSF) circulatory system and can be visualized by MRI. MRI-assisted measurement of ONSASW is a significant method for non-invasive quantitative estimation of CSF pressure [34]. The results also suggest that the cerebral spinal fluid pressure (CSFP) in the CRVO-affected eyes group was higher than that in the BRVO group. Our results indicate that the CSFP in the CRVO-affected eyes group is higher than that in the contralateral normal eyes and the BRVO-affected eyes group.

Wang et al. reported that the formula to calculate the level of trans-lamina cribrosa pressure difference (TLCPD) was IOP-CSFP [6]. TLCPD was a better marker for glaucoma than IOP [35]. In our study, the proportion of the higher IOP was significantly higher in the CRVO-affected eyes group than that in the BRVO-affected eyes group (24% vs. 6%), indicating that the CSFP in the CRVO-affected eyes group was also higher than that in the BRVO-affected eyes group. It was reported that the higher TLCPD in subjects with higher IOP is an independent risk factor for glaucoma [36]. Higher intracranial pressure may prevent the progression of primary open-angle glaucoma [37]. Our study found that CRVO may be associated with open-angle glaucoma, consistent with previous reports that there was a closer association between glaucoma and CRVO than BRVO [5].

Fundus photography (FP) has been a quick, simple, non-invasive, and commonly used diagnostic procedure for detecting retinal disorders and systemic vascular diseases. FP is easy to use, cost-effective, more adaptable, and accepted by doctors in other disciplines. FP has been a bridge for collaborations between ophthalmologists and endocrinologists or neurologists. Although OCT, especially OCTA with higher resolution, has been a popular examination tool in eye clinics, FP is more suitable for studying the correlation between retinal vascular changes and systemic diseases. Multi-modal imaging could provide a complete description of the microvascular and structural alterations; developing an AI-based algorithm using multimodal images, including FP, OCT/OCTA, autofluorescence, etc., is warranted [38,39].

Furthermore, Castro et al. found that the agreement between multimodal imaging (infrared, blue reflectance, and green reflectance monochromatic and OCT) and color fundus photography was substantial to almost perfect for most lesions in RVO. Multi-modal imaging seems better for detecting venous sheathing in RVO. Opto-cilliary shunts seem easier to detect on FP [40].

The limitation of this study is that this is a case-control study, the causal relationship between retinal vascular caliber and cerebrovascular diseases cannot be made, and a large sample size with a well-designed cohort is warranted in the future. ONSASW is an invasive method to indirectly evaluate the CSF-P. A more convenient method that was developed in recent years, such as B ultrasound, has been shown to have the privilege of providing an accurate evaluation of CSF by calculating the area (not the width) of the orbital optic nerve subarachnoid space. With continuous revolutionary techniques in the near future, the correlation between CSF-P and POAG can be elucidated more clearly in the pathogenesis of RVO.

## 5. Conclusions

We concluded that BRVO may be more associated with cerebrovascular diseases, and CRVO may be correlated with primary angle glaucoma. The geometric characteristics difference between the retinal and cerebrovascular may explain the pathological difference between CRVO and BRVO. BRVO and CRVO are otherwise distinct entities, emphasizing that various management approaches should be considered in clinical practice. The geometric characteristics can be used as imaging biomarkers for BRVO and CRVO.

## Figures and Tables

**Table 1:** Demographic characteristics of all the enrolled subjects.

	Subjects with BRVO (Eyes)	Subjects with CRVO (Eyes)	t/x^2^/Z	*p* Value
	34 (68)	25 (50)		
Age, y, (mean ± SD)	53 ± 10	50 ± 15	0.782 ^a^	0.439
Gender, male/female, *n*	18/16	16/9	0.722 ^b^	0.396
Height, m, (mean ± SD)	1.65 ± 0.06	1.66 ± 0.08	−0.124 ^a^	0.902
Weight, kg, (mean ± SD)	71.12 ± 9.43	71.43 ± 9.27	−0.115 ^a^	0.909
BMI, kg/m^2^, (mean ± SD)	25.97 ± 2.47	26.08 ± 3.04	−0.150 ^a^	0.882
Duration of HBP, y, (IQR)	0.0 (0.0–4.5)	0.0 (0.0–0.0)	−2.298 ^c^	0.022 *
IOP, (IQR)				
Affected eyes (IQR)	16.75 (13.98–17.68)	17.50 (15.70, 21.30)	−2.264 ^c^	0.024 *
Contralateral normal eyes (IQR)	16.00 (13.15, 19.05)	16.00 (14.50, 17.00)	−0.142 ^c^	0.887

^a^ Independent sample *t*-test. ^b^ Chi-square test. ^c^ Mann-Whitney U test. BRVO: branch retinal vein occlusion; CRVO: central retinal vein occlusion; SD: standard deviation; IQR: interquartile range; BMI: body mass index; HBP: high blood pressure; IOP: intraocular pressure. * Statistically significant: *p* < 0.05.

**Table 2:** Comparison of CBVGCs and RVGCs between affected and contralateral eyes in subjects with BRVO or CRVO.

	Mean ± SD				Paired Sample Test (*p* Value) (Affected Eyes vs. Contralateral Eyes)		Independent-Sample *t* Test (*p* Value) (BRVO vs. CRVO)	
	BRVO-Affected Eyes	BRVO-Contralateral Normal Eyes	CRVO-Affected Eyes	CRVO-Contralateral Normal Eyes	BRVO	CRVO	Affected Eyes	Contralateral Normal Eyes
IOP, mmHg, (mean ± SD)	16.39 ± 3.00	16.11 ± 3.79	19.58 ± 8.84	15.92 ± 1.74	0.525 ^a^	0.027 ^b,^*	0.229 ^c^	0.802 ^c^
The diameter of the ICA-C6, mm, (mean ± SD)	4.07 ± 0.61	4.03 ± 0.51	3.98 ± 0.50	3.91 ± 0.50	0.748 ^a^	0.353 ^b^	0.584 ^c^	0.363 ^c^
The diameter of the OA, mm, (mean ± SD)	3.23 ± 0.43	3.22 ± 0.46	3.40 ± 0.41	3.47 ± 0.28	0.862 ^a^	0.488 ^a^	0.153 ^c^	0.014 ^c,^*
ONSASW-3, mm, (mean ± SD)	1.01 ± 0.21	0.99 ± 0.34	1.16 ± 0.19	1.01 ± 0.16	0.763 ^a^	0.003 ^b,^*	0.001 ^d,^*	0.029 ^d,^*
ONSASW-9, mm, (mean ± SD)	0.86 ± 0.15	0.82 ± 0.12	1.01 ± 0.21	0.88 ± 0.14	0.103 ^a^	0.015 ^b,^*	0.022 ^d,^*	0.116 ^c^
ONSASW-15, mm, (mean ± SD)	0.79 ± 0.12	0.78 ± 0.13	0.84 ± 0.10	0.81 ± 0.09	0.784 ^a^	0.110 ^a^	0.063 ^d^	0.366 ^c^
The relative retinal arteriolar caliber, (mean ± SD)	0.057 ± 0.010	0.058 ± 0.010	0.059 ± 0.015	0.062 ± 0.009	0.272 ^a^	0.229 ^b^	0.453 ^d^	0.191 ^c^
The relative retinal venular caliber, (mean ± SD)	0.076 ± 0.009	0.077 ± 0.010	0.095 ± 0.015	0.077 ± 0.011	0.731 ^b^	<0.001 ^a,^**	<0.001 ^d,^*	0.851 ^c^
AVR, (mean ± SD)	0.747 ± 0.088	0.766 ± 0.107	0.642 ± 0.125	0.809 ± 0.076	0.188 ^b^	<0.001 ^a,^**	0.001 ^d,^*	0.098 ^c^
Retinal vascular branching angles, (mean ± SD)								
Retinal superior nasal artery (mean ± SD)	62.406 ± 14.605	63.482 ± 16.364	48.994 ± 12.865	62.583 ± 24.240	0.732 ^a^	0.037 ^b,^*	0.001 ^c,^*	0.867 ^c^
Retinal superior nasal vein	66.999 ± 19.078	61.722 ± 18.242	55.403 ± 20.259	61.500 ± 18.108	0.177 ^a^	0.252 ^a^	0.032 ^c,^*	0.963 ^c^
Retinal inferior nasal artery	64.673 ± 19.888	65.773 ± 16.094	53.089 ± 16.113	59.049 ± 14.238	0.805 ^a^	0.175 ^a^	0.020 ^c,^*	0.104 ^c^
Retinal inferior nasal vein	67.788 ± 16.344	65.380 ± 20.733	59.778 ± 19.382	69.518 ± 14.393	0.438 ^a^	0.071 ^a^	0.094 ^c^	0.397 ^c^
Retinal superior temporal artery	68.421 ± 18.789	71.354 ± 11.650	56.155 ± 16.306	65.832 ± 14.445	0.484 ^a^	0.010 ^a,^*	0.011 ^c,^*	0.113 ^c^
Retinal superior temporal vein	66.719 ± 15.836	67.096 ± 16.945	66.907 ± 23.359	64.552 ± 17.940	0.989 ^a^	0.762 ^a^	0.971 ^c^	0.583 ^c^
Retinal inferior temporal artery	78.268 ± 25.170	74.581 ± 17.619	57.872 ± 22.942	80.617 ± 21.042	0.570 ^a^	<0.001 ^a,^**	0.002 ^c,^*	0.240 ^c^
Retinal inferior temporal vein	72.225 ± 21.063	74.826 ± 16.625	67.992 ± 23.633	66.566 ± 17.837	0.560 ^a^	0.826 ^a^	0.472 ^c^	0.075 ^c^

^a^ Paired sample *t*-test. ^b^ Wilcoxon signed-rank test. ^c^ Independent-sample *t* test. ^d^ Mann-Whitney U test. BRVO: branch retinal vein occlusion; CRVO: central retinal vein occlusion; CBVGCs: cerebral blood vessel geometric characteristics; RVGCs: retinal vessel geometric characteristics; SD: standard deviation; IOP: intraocular pressure; ICA-C6: ophthalmic (C6) segment of the internal carotid artery; OA: ophthalmic artery; ONSASW: optic nerve subarachnoid space width; AVR: arteriolar-to-venular diameter ratio. * Statistically significant: *p* < 0.05. ** Statistically significant: *p* < 0.001.

## Data Availability

Data underlying the results presented in this paper are not publicly available at this time but may be obtained from the authors upon reasonable request.

## Abbreviations

AI	Artificial intelligence
AVR	Arteriolar-to-venular diameter ratio
BRVO	Branch retinal vein occlusion
BMI	Body mass index
CRVO	Central retinal vein occlusion
CRA	Central retinal artery
CRV	Central retinal vein
CBVGCs	Cerebral blood vessel geometric characteristics
COAG	Chronic open-angle glaucoma
CSF-P	Cerebrospinal fluid pressure
EDV	End-diastolic velocity
FFA	Fundus fluorescein angiography
FRFSE	Fast recovery, fast spin echo
HBP	Hypertension
HRVO	Hemispheric retinal vein occlusion
HU	Hounsfield units
ICA	Internal carotid artery
IOP	Intraocular pressure
MRI	Magnetic resonance imaging
MRA	Magnetic resonance angiography
OA	Ophthalmic artery
OCT	Optical coherence tomography
ONSASW	Optic nerve subarachnoid space width
OR	Odd ratio
PCA	Posterior ciliary artery
POAG	Primary open-angle glaucoma
PPP	Preferred Practice Patterns
RVO	Retinal vein occlusion
RVGCs	Retinal vessel geometric characteristics
VEGF	Vascular endothelial growth factor
